# Supplementary material for: The Systems Biology Research Tool: evolvable open-source software
Source: BMC Syst Biol. 2008 Jun 29;2:55. doi: 10.1186/1752-0509-2-55 (PMC2446383; doi:10.1186/1752-0509-2-55)
Supplement: Additional file 1 — SBRT Archive. An archive of the current version of the Systems Biology Research Tool. [file 1752-0509-2-55-S1.zip › sbrt-1.4.0/doc/developers_guide/api/sbrt/kernel/util/class-use/Or.html]

Uses of Class sbrt.kernel.util.Or


|  |  |  |
| --- | --- | --- |
| **Overview** \| **Package** \| **Class** \| **Use** \| **Tree** \| **Deprecated** \| **Index** \| **Help** | |  |
| PREV   NEXT | **FRAMES**    **NO FRAMES**     **All Classes** |  |


---


## **Uses of Class sbrt.kernel.util.Or**

| Packages that use Or | |
| --- | --- |
| **sbrt.kernel.fba** | Provides classes and interfaces for creating objects relevant to Flux Balance Analysis. |
| **sbrt.shell.mng.fba** | Provides classes and interfaces for creating Flux Balance Analysis process managers. |
| **sbrt.shell.text** | Provides classes and interfaces for defining formats for basic objects. |
| **sbrt.shell.text.fba** | Provides classes and interfaces for defining formats for objects relevant to Flux Balance Analysis. |

Uses of Or in sbrt.kernel.fba

| Methods in sbrt.kernel.fba that return Or | |
| --- | --- |
| `Or<And<java.lang.String>>` | `IrrevRxnFluxome.getCatalystRule(java.lang.String rxnName)`             Returns the boolean logic statement for the catalysts of the reaction with the provided name. |
| `Or<And<java.lang.String>>` | `IrrevRxnFluxomeV1.getCatalystRule(java.lang.String rxnName)`             Returns the boolean logic statement for the catalysts of the reaction with the provided name. |

| Methods in sbrt.kernel.fba that return types with arguments of type Or | |
| --- | --- |
| `java.util.Map<java.lang.String,Or<And<java.lang.String>>>` | `IrrevRxnFluxome.getCatalystRules()`             Returns all of the boolean logic statements regarding reaction catalysis. |
| `java.util.Map<java.lang.String,Or<And<java.lang.String>>>` | `IrrevRxnFluxomeV1.getCatalystRules()`             Returns all of the boolean logic statements regarding reaction catalysis. |

Constructor parameters in sbrt.kernel.fba with type arguments of type Or

`IrrevRxnFluxomeV1(java.util.Map<java.lang.String,ChemRxn> rxns,
java.util.Map<java.lang.String,Or<And<java.lang.String>>> catalysts)`
  
          Constructs a new irreversible reaction fluxome from the
provided internal and exchange reactions and catalysts.

`IrrevRxnFluxomeV1(java.util.Map<java.lang.String,IrrevRxn> internalRxns,
java.util.Map<java.lang.String,SourceRxn> sourceRxns,
java.util.Map<java.lang.String,SinkRxn> sinkRxns,
java.util.Map<java.lang.String,Or<And<java.lang.String>>> catalysts)`
  
          Constructs a new irreversible reaction fluxome from the
provided internal and exchange reactions and catalysts.

Uses of Or in sbrt.shell.mng.fba

| Methods in sbrt.shell.mng.fba that return types with arguments of type Or | |
| --- | --- |
| `static java.util.Map<java.lang.String,Or<And<java.lang.String>>>` | `FbaManager.getCatalysts(Fluxome fluxome, java.lang.String fileName)`             Returns the reaction-catalyst associations contained in the file with the provided name. |
| `static java.util.Map<java.lang.String,Or<And<java.lang.String>>>` | `FbaManager.getCatalysts(Fluxome fluxome, java.lang.String fileName, java.io.PrintWriter out)`             Returns the reaction-catalyst associations contained in the file with the provided name. |

Uses of Or in sbrt.shell.text

| Methods in sbrt.shell.text that return Or | |
| --- | --- |
| `Or<And<E>>` | `BoolRelFormatV1.parse(java.lang.String set)`             Parses the provided string and returns its corresponding collection of elements as a `LinkedHashSet`. |

| Methods in sbrt.shell.text with parameters of type Or | |
| --- | --- |
| `java.lang.String` | `BoolRelFormatV1.format(Or<And<E>> boolRel)`             Returns a formatted string representation of the provided boolean relationship. |

Uses of Or in sbrt.shell.text.fba

| Methods in sbrt.shell.text.fba that return Or | |
| --- | --- |
| `Or<And<java.lang.String>>` | `PalssonRxnNodeParser.getCatalysts(org.w3c.dom.Node reactionNode, java.lang.String prefix)`             Returns the boolean logic rules for the catalysts of the provided reaction node. |
| `Or<And<java.lang.String>>` | `BiggRxnNodeParser.getCatalysts(org.w3c.dom.Node reactionNode, java.lang.String prefix)`             Returns the boolean logic rules for the catalysts of the provided reaction node. |
| `Or<And<java.lang.String>>` | `PalssonRxnNodeParser.getGenes(org.w3c.dom.Node reactionNode)`             Calls PalssonRxnNodeParser.getCatalysts(Node, String) with "`GENE_ASSOCIATION:`" as the `prefix`. |
| `Or<And<java.lang.String>>` | `BiggRxnNodeParser.getGenes(org.w3c.dom.Node reactionNode)`             Calls BiggRxnNodeParser.getCatalysts(Node, String) with "`GENE ASSOCIATION:`" as the `prefix`. |
| `Or<And<java.lang.String>>` | `PalssonRxnNodeParser.getProteins(org.w3c.dom.Node reactionNode)`             Calls PalssonRxnNodeParser.getCatalysts(Node, String) with "`PROTEIN_ASSOCIATION:`" as the `prefix`. |
| `Or<And<java.lang.String>>` | `BiggBracketParserV1.parse(java.lang.String string)`             Parses the provided BiGG style bracketed expression. |

---


|  |  |  |
| --- | --- | --- |
| **Overview** \| **Package** \| **Class** \| **Use** \| **Tree** \| **Deprecated** \| **Index** \| **Help** | |  |
| PREV   NEXT | **FRAMES**    **NO FRAMES**     **All Classes** |  |


---
